# Ketamine for pain management

**DOI:** 10.1097/PR9.0000000000000674

**Published:** 2018-08-09

**Authors:** Rae Frances Bell, Eija Anneli Kalso

**Affiliations:** a Regional Centre of Excellence in Palliative Care, Haukeland University Hospital, Bergen, Norway; b Perioperative, Intensive Care and Pain Medicine, University of Helsinki and Pain Clinic, Helsinki University Hospital, Helsinki, Finland

**Keywords:** Ketamine, Pain management

## Abstract

Supplemental Digital Content is Available in the Text.

**Key Points**

1. There is good evidence that perioperative ketamine decreases postoperative pain scores and opioid requirements, but there is a lack of consensus on dose, for both bolus and infusion.
2. Despite limited evidence, a trial of low-dose intravenous or subcutaneous ketamine adjuvant to morphine may be warranted in refractory cancer pain.
3. There is only very limited evidence for the use of ketamine in chronic noncancer pain and concerns and a lack of safety data concerning long-term or repeated treatment. Importantly, there is no strong evidence to support the current practice of treating chronic noncancer pain with repeated intravenous infusions.
4. Ketamine has dose-dependent adverse effects, and there are good arguments for avoiding high doses.
5. Spinal administration is associated with neurotoxicity, whereas oral ketamine has low bioavailability and is associated with adverse effects.

## 1. Background

### 1.1. Basic pharmacology and mechanisms of action of ketamine

Ketamine is commonly used as an analgesic in emergency medicine and as an adjuvant drug in the perioperative setting. In addition, it is used as a third-line adjuvant drug for opioid-resistant pain in palliative care and for intractable chronic noncancer pain. More recently, ketamine is increasingly being used to treat major depression and other mood disorders.

Ketamine is a phencyclidine derivative that was developed in the 1960s as an anaesthetic agent. The most important pharmacological properties of ketamine are due to it being a noncompetitive *N*-methyl-d-aspartate (NMDA) receptor antagonist, and its analgesic action at subanaesthetic dose is believed to be primarily due to NMDA receptor antagonism in the brain and spinal cord.^33^ The NMDA receptor is important for learning, memory, and synaptic plasticity. Regarding pain, the NMDA receptor is involved in the amplification of pain signals, the development of central sensitization, and opioid tolerance.^49^ Ketamine has been shown to have antihyperalgesic effects and to reduce or reverse opioid tolerance.^22,24^

Ketamine also interacts with other receptors and channels, including nicotinic and muscarinic acetylcholine receptors, opioid receptors, monoaminergic receptors, and voltage-sensitive sodium channels.^41^ It enhances endogenous antinociceptive systems, increasing the descending inhibitory serotoninergic pathway.^33^ Recent research indicates that ketamine may also modulate (suppress) pain transmission by limiting astrocyte and microglial activation.^46^

Ketamine gives robust and rapid relief of major depression and suicidal ideation.^1^ The mechanism for this effect is as yet not fully elucidated, but major depressive disorder is associated with synaptic downregulation in the prefrontal cortex and hippocampus, and it is believed that ketamine causes a glutamate surge that leads to a series of events resulting in synaptogenesis and reversal of the negative effects of depression and chronic stress.^1^ A recent functional magnetic resonance imaging study in patients with treatment-resistant depression compared with healthy volunteers demonstrated that ketamine normalized depression-related prefrontal dysconnectivity.^2^

Ketamine has anti-inflammatory effects, modulating the production of different proinflammatory mediators. A recent study using a rabbit model of gonarthrosis found that ketamine suppressed the inflammatory response in osteoarthritis,^28^ whereas a systematic review concluded that intraoperative ketamine reduces the postoperative IL-6 inflammatory response in surgical patients.^15^

The most commonly reported adverse effects of ketamine are psychotomimetic (hallucinations, agitation, anxiety, dysphoria, and euphoria). Administration of ketamine may also cause dizziness, nausea, sedation, and tachycardia. Ketamine's adverse effects are dose-dependent. At low dose, it acts as an NMDA receptor antagonist, providing an analgesic effect; but at higher doses, it acts on other receptors and channels including dopamine D_2_ receptors, monoaminergic receptors, and opioid receptors.^62^ Ketamine dose dependently inhibits monoamine transporters, and it has been suggested that this may be one mechanism behind its psychotomimetic adverse effects.^41^ Chronic abuse of ketamine is associated with a range of adverse effects, some of which have also been reported in pain patients treated with ketamine (Tables 1 and 2).

**Table 1:**
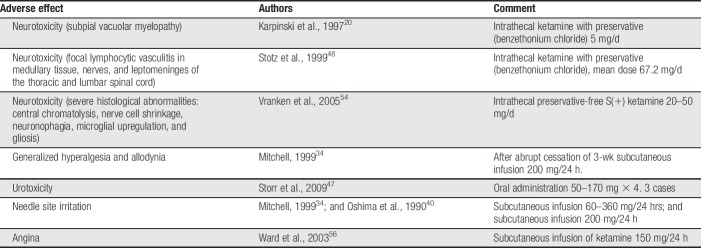
Adverse effects of ketamine, other than psychotomimetic, reported in cancer/palliative care pain management.

**Table 2:**
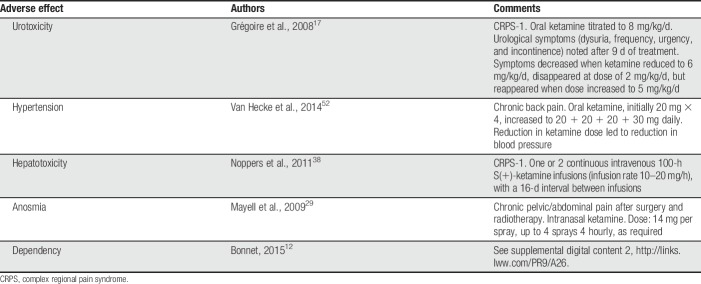
Adverse effects of ketamine, other than psychotomimetic, reported in chronic noncancer pain management.

### 1.2. Pharmacokinetics and administration of ketamine

Ketamine is available as a racemic mixture or as the S(+) enantiomer that is approximately twice as potent as racemic ketamine and about 4 times as potent as the R(−) enantiomer.^33^ Ketamine is N-demethylated by liver microsomes into the major metabolite of racemic ketamine, norketamine, which is rapidly metabolized to ketamine's major secondary metabolite, 6-hydroxynorketamine, and to the lesser metabolites 4-hydroxyketamine and 6-hydroxyketamine.^33^ Ketamine has been shown to have antinociceptive effects and to reduce opioid tolerance, whereas norketamine has been shown to have antinociceptive properties in animal studies, but little is known about its analgesic effects in humans. A study in healthy volunteers found that S(+)-norketamine after S(+) ketamine dosing did not have significant antinociceptive properties and even made a negative contribution to S(+) ketamine analgesia.^39^ Six-hydroxynorketamine in an animal model failed both to demonstrate antinociceptive properties and to attenuate opioid tolerance.^26^

Ketamine may be given by multiple routes of administration including intravenous (IV), subcutaneous, oral, intranasal, transdermal, and spinal (epidural and intrathecal). Spinal administration of ketamine has been shown to be associated with neurotoxicity, safety data are lacking and no NMDA receptor antagonists have been approved for neuraxial use in humans.^57^ The oral bioavailability of ketamine is low, reportedly 17% to 24% for oral racemic ketamine and 8% to 11% for oral S(+)-ketamine.^41^ After oral intake of ketamine, norketamine plasma concentrations are much higher than those of the parent drug.^41^

Ketamine does not cause respiratory depression and does not decrease blood pressure, making it a useful drug in emergency medicine and intensive care, although there are certain concerns because of its effect on intracranial pressure and ocular pressure. The use of ketamine in emergency medicine and intensive care is beyond the scope of this review.

Ketamine's antihyperalgesic, antidepressant, and anti-inflammatory effects, together with its beneficial interactions with opioids, including reduction of opioid tolerance, make it an especially interesting drug to use in pain management. However, depending on the clinical setting, the extent to which ketamine is clinically useful depends not only on its efficacy and tolerability but also on other factors such as ease of administration, costs, and long-term safety.

The best evidence for efficacy and tolerability comes from systematic reviews of randomized controlled trials (RCTs), whereas case reports provide valuable information on adverse effects. In this review, we will examine the current evidence for the benefits and harms of ketamine and its clinical usefulness in pain management.

## 2. Ketamine for acute postoperative pain

There is a large body of literature addressing the use of ketamine in the perioperative setting. Adjuvant treatment with IV racemic, or S(+) ketamine is common, to improve postoperative pain relief and reduce opioid requirements. Epidural ketamine has also been used in this setting. In some cases, ketamine has been used with the aim of preventing chronic postoperative pain.

### 2.1. Evidence for efficacy and adverse effects

A search of PubMed on February 23, 2018, using the terms “ketamine” AND “postoperative pain” and the filter “systematic reviews” yielded 56 hits, 11 of which were systematic reviews addressing perioperative ketamine for acute or chronic postoperative pain^4,8,16,21,30,36,42,55,59,60,63^ (Table 3). A recently completed Cochrane review,^14^ currently under review, has also been included.

**Table 3:**
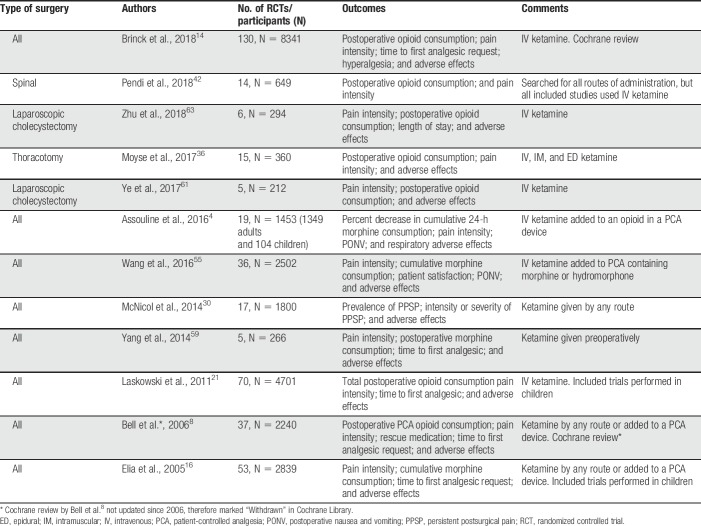
Systematic reviews of ketamine for postoperative pain.

Perioperative ketamine decreased postoperative pain scores,^4,8,16,21,30,36,42,55,60,63^ increased the time to first analgesic request,^21,60^ and reduced postoperative opioid requirements.^4,8,16,21,42,55,59,60,63^ Ketamine also reduced postoperative nausea and/or vomiting.^4,21,36,55,60,63^ Interestingly, Laskowski et al.^21^ found that ketamine had significant analgesic benefit for major procedures involving the upper abdomen and thorax, associated with high pain scores or high opioid requirements. This finding was also reported by the most recent Cochrane review.^14^

One review that focused on ketamine for persistent postsurgical pain^30^ did not find an overall reduced risk of developing chronic postoperative pain in the ketamine group compared with placebo. However, when exclusively IV ketamine studies were analysed, they demonstrated statistically significant risk reduction at 3 and 6 months. According to the authors, this could have been due to spinal NMDA receptors playing only a minor role as targets for ketamine in postoperative pain, or due to ketamine having lower systemic efficacy when it is administered by the epidural route.

Most systematic reviews report that perioperative ketamine was well tolerated, with adverse effects being mild or absent. Most studies provided dichotomous data on central nervous system adverse events.^14^

#### 2.1.1. Clinical considerations

Although most trials investigated ketamine given by the IV route, clinical regimens differed, especially regarding dose. Data from the review by Brinck et al.^14^ indicate that 77 of the 130 trials used racemic ketamine as an IV bolus, with 35 of these studies using a dose less than 0.25 mg/kg. Twenty-one studies used a bolus dose of 0.5 to 1 mg/kg. Forty-two trials used racemic ketamine as a continuous IV infusion, with the most common dose being 2 to 5 μg/kg/min. Ten trials used S(+) ketamine, of these 8 used a preincisional IV bolus (0.075–0.5 mg/kg), followed by an IV infusion (0.25–6.7 μg/kg/min). One trial used R(−) ketamine as an IV bolus (1 mg/kg).

Ketamine seems most beneficial when pain scores are high, suggesting that it is primarily useful for surgery associated with high levels of postoperative pain. Given that ketamine reduces opioid requirements, it may also be indicated for subgroups such as opioid tolerant or opioid-dependent patients.

## 3. Ketamine for opioid-resistant pain in palliative care

Ketamine is widely used as a third-line drug for cancer pain, when the pain has not responded to opioid in combination with drugs such as nonsteroidal anti-inflammatory drugs, amitriptyline, and gabapentinoids. Published case reports demonstrate that ketamine is used for refractory pain in palliative care in many countries, and that treatment regimens differ widely using IV, subcutaneous, oral, intrathecal, and topical routes of administration. A Cochrane review on ketamine as an adjuvant to opioid for cancer pain,^9^ first published in 2003, described 32 case reports involving 246 patients treated with ketamine. Doses ranged from 1 mg/kg/d as a subcutaneous infusion, to 600 mg/d as an IV infusion and 67.2 mg/d intrathecally.

### 3.1. Evidence for efficacy and adverse effects

A simple search of PubMed using the terms “ketamine” AND “cancer” and the filter “systematic reviews” yielded 14 hits, 4 of which were systematic reviews on ketamine for cancer pain. Three of the 4 titles were different versions of the Cochrane review on ketamine as an adjuvant to opioid in cancer pain in adults, which has been twice updated, most recently in 2017.^9^ The fourth title was a comprehensive review of all available English language literature on ketamine for cancer pain in children and adults^13^ (Table 4).

**Table 4:**
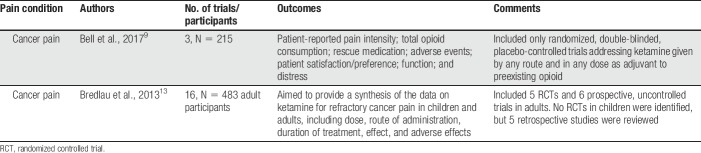
Systematic reviews on ketamine for refractory cancer pain.

The recently updated Cochrane review included 3 RCTs. Two small cross-over trials investigated IV ketamine in 2 doses^31^ or intrathecal ketamine^58^ as an adjuvant to morphine. Both trials found that ketamine reduced pain intensity and morphine requirements. A third larger trial with a parallel-group design and 185 participants investigated rapid titration of subcutaneous ketamine to high dose (500 mg) in participants who were using different opioids.^18^ In this trial, there was no difference between groups regarding patient-reported pain intensity, and there was almost twice the incidence of adverse effects in the ketamine group. Two serious adverse events (bradyarrhythmia and cardiac arrest) believed to be related to ketamine were reported in this trial. The update concluded that current evidence is insufficient to assess the benefits and harms of ketamine as an adjuvant to opioids for refractory cancer pain, and that rapid dose escalation of ketamine to high dose (500 mg) does not seem to have clinical benefit and may be associated with serious adverse events.

The review by Bredlau et al.,^13^ which used a comprehensive approach and less stringent methodology than the Cochrane review included 5 RCTs and 6 prospective uncontrolled trials on ketamine for cancer pain in adults. No RCTs in children were identified, but the authors considered 5 retrospective studies. The review found that ketamine reduced opioid requirements, and may improve pain control, at the same time noting the significant limitations of the current evidence and the wide variation in dosages, routes, duration, and frequency of ketamine administration in the published literature. The authors also expressed concerns regarding ketamine's neurotoxic effects, recommending that intrathecal administration and epidural administration should be avoided. The review concludes that “*In children and adults with cancer pain that has not responded adequately to standard therapy, the literature supports considering ketamine as an adjuvant therapy*.”

Several reports have described neurotoxicity when ketamine was administered intrathecally in the palliative care setting.^20,48,54^ Abrupt cessation of a continuous subcutaneous infusion was reported to result in generalized hyperalgesia.^34^ Storr et al. reported 3 patients treated by a palliative care team who developed urological symptoms (frequency, haematuria, dysuria, and bladder pain) after oral ketamine.^47^ For reports of adverse effects of ketamine, other than psychotomimetic, in the management of refractory cancer pain, see Table 1.

#### 3.1.1. Clinical considerations

We concur with the review by Bredlau et al.^13^ that although the evidence is limited, ketamine may be useful as a third-line drug for selected patients with refractory cancer pain. Many of the case studies describe a dramatic effect of ketamine, but as yet, we are unable to identify characteristics of responders. The choice of opioid may be of importance because recent animal studies have shown that ketamine and norketamine attenuate morphine tolerance more effectively than oxycodone tolerance.^25^

There seem to be good arguments for keeping the ketamine dose low. Ketamine has dose-dependent adverse effects. Terminally ill cancer patients may have reduced hepatic function because of metastases and diminished liver perfusion. Hepatic impairment can cause reduced drug metabolism and significantly impaired clearance.^37^ Increased age has also been shown to be associated with substantially reduced ketamine clearance.^23^ Examples of low-dose regimens adjuvant to opioid, primarily morphine, are racemic ketamine 1 mg/kg/24 hours per day,^7^ or S(+) ketamine (0.5–2 mg/kg/24 hours) as an IV infusion, with careful individual titration.^10^

The evidence for efficacy and tolerability for ketamine in this setting is limited. It is undeniably challenging to conduct RCTs in this patient group. What then in theory could be the indications for ketamine treatment? When cancer pain or pain in a palliative care patient is refractory to opioid and adjuvant drugs, then ketamine may be an option. There are many reasons for pain in this patient group. Ketamine could be especially relevant when there are problems of opioid tolerance, a significant neuropathic pain component, inflammatory pain, depression, or any combination of these factors. In fact, this may be the pain patient group that is most likely to need a trial of ketamine and where the risk benefit ratio is the most beneficial. A trial of ketamine does not need to be lengthy and if there is no clear benefit, then ketamine treatment should be terminated.

## 4. Ketamine for chronic noncancer pain

It is more difficult to formulate clear indications for the use of ketamine in chronic noncancer pain. Given ketamine's range of adverse effects and the lack of safety data concerning long-term treatment, there is good reason to be cautious when treating complex chronic pain problems with this drug. Possible indications could be refractory neuropathic or inflammatory pain with, or without, depression in carefully selected patients.

Ketamine is increasingly being used as a third-line drug for refractory chronic noncancer pain. In this setting, it is commonly administered as intermittent IV infusions. Patients may be offered hospital admission and infusion treatment over several days. However, outpatient treatment seems to be on the increase, and in the United States, a large number of “ketamine clinics” have been established offering infusions for a variety of conditions, including chronic pain, depression, and other mood disorders. A Medscape report suggests that there may be more than 1000 such clinics currently operating in the United States.^51^ For chronic pain, these clinics offer a series of infusions on an outpatient basis, followed by “maintenance therapy” for example, involving monthly ketamine infusions.^5^ Although racemic ketamine is an inexpensive drug, patient costs associated with this treatment are high (supplemental digital content 1, http://links.lww.com/PR9/A25).^5^

### 4.1. Evidence for efficacy and adverse effects

A search of PubMed on February 23, 2018, using the terms “ketamine” AND “chronic pain” and the filter “systematic reviews” yielded 20 hits, of which 4 were systematic reviews on ketamine for chronic noncancer pain in adults^6,19,32,61^ (Table 5).

**Table 5:**
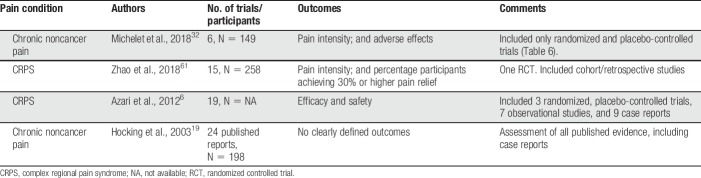
Systematic reviews of ketamine for chronic noncancer pain.

Of the 2 reviews published in 2018, Michelet et al.^32^ included only randomized, placebo-controlled, double-blinded trials on ketamine for chronic pain in adults (Table 6). The authors found low-level evidence (GRADE) demonstrating that ketamine was ineffective regarding the primary outcome of the review, failing to decrease pain intensity at 4 weeks after the beginning of treatment. When only trials not judged to have a high risk of bias were analyzed, they found moderate-level evidence that ketamine was effective at 4 weeks after treatment, suggesting a long-lasting effect. However, the clinical implications of this finding are uncertain because trial sequential analysis found the meta-analysis to be underpowered and methodological shortcomings in several of the included trials have been noted. The authors judged the trials by Mitchell et al.^35^ and Amr^3^ to be at high risk of bias, whereas the methodology and conclusions of the trial by Schwartzman et al.,^44^ which was prematurely terminated before even half of the planned number of patients had been included, have also been challenged.^11^ The carefully performed trial by Sigtermans et al.^45^ found that ketamine infusions gave pain relief for patients with complex regional pain syndrome (CRPS) type I but did not result in functional improvement. As the authors pointed out, blinding in this trial was possibly compromised by ketamine-related adverse effects. Follow-up times in these chronic pain RCTs were 1 week,^53^ 9 days,^35^ 11 weeks,^45^ and 3 months.^43,44^

**Table 6:**
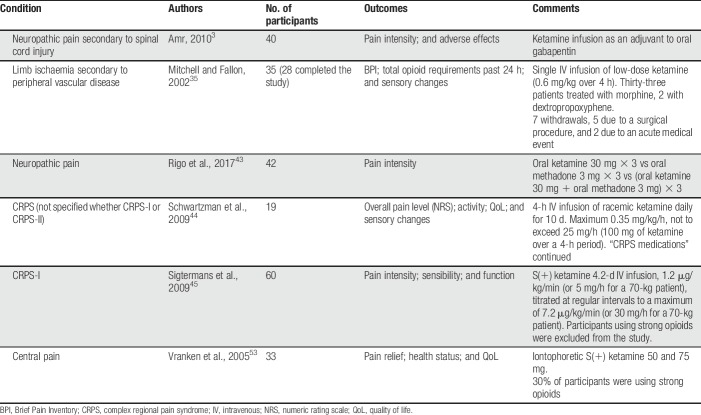
Randomized, placebo-controlled, double-blinded trials on ketamine for chronic pain in adults (Michelet et al., 2018^32^).

Ketamine was generally associated with more adverse effects than placebo. The studies were heterogenous regarding dose, route of administration, and duration of treatment, and the authors of the review were unable to recommend any treatment regimen, noting the need for further trials.

Zhao et al.^61^ addressed ketamine infusions for CRPS-I and CRPS-II and analyzed data from 258 participants in 15 trials. However, only one of the 15 included studies was an RCT, and retrospective studies were also included. Fourteen of the 15 studies used S(+) ketamine infusions, whereas one used racemic ketamine. The authors concluded that that ketamine may provide short-term pain relief, but that further studies are required to confirm this conclusion.

#### 4.1.1. Clinical considerations

Sigtermans et al.^45^ when discussing the lack of functional improvement in the ketamine group speculated whether more prolonged treatment, possibly in combination with physical therapy or rehabilitation strategies, could be necessary. Combining ketamine treatment with rehabilitation strategies is an important aspect, which as yet, has not been addressed by any RCT.

The addiction literature contains numerous reports on the neurotoxic, hepatotoxic, and urotoxic adverse effects of ketamine. There are also case reports concerning similar ketamine-related adverse effects in patients with chronic pain (Table 2). Grégoire et al.^17^ described cystitis in a 16-year-old patient treated for CRPS-I with oral ketamine. A patient with chronic back pain developed uncontrolled hypertension (blood pressure 224/124 mm Hg), 1 week after starting treatment with oral ketamine.^52^ Noppers et al.^38^ described 3 of 6 CRPS-I patients treated with ketamine who developed hepatotoxicity after S(+)-ketamine infusions. A patient with chronic pain after cancer therapy and with no detected recurrence who received treatment with intranasal ketamine developed permanent anosmia 6 months after starting treatment.^29^

Liu et al.^27^ have recently reported ketamine-related upper gastrointestinal (GI) tract toxicity, such as epigastric pain (“K-cramps,” “K-belly”), vomiting, anaemia, and GI bleeding, in 25% of inhalational abusers of ketamine seeking treatment for urotoxicity, and advise that young patients presenting with upper GI symptoms should be questioned about the recreational use of ketamine. It is not common knowledge that repeated administration of ketamine may cause GI symptoms. Whether such symptoms could arise in relation to repeated infusions of ketamine is unknown, but there is a possibility that ketamine-related GI toxicity could be overlooked in the clinical setting.

Ketamine's potential for addiction should also be considered when patients are treated with intermittent IV infusions. Ketamine is a popular club drug. In animal models, repeated administration in subanaesthetic doses causes sensitisation, a characteristic of drugs such as cocaine. Trujillo found the results of his rodent studies to be sufficiently concerning as to advise caution regarding the repeated use of ketamine, both recreationally and in the clinical setting.^50^ Bonnet^12^ report a patient initially treated for back pain and recurrent depression who subsequently abused and developed an addiction to ketamine (supplemental digital content 2, http://links.lww.com/PR9/A26).

Because ketamine's adverse effects are dose-dependent, high doses should be avoided. Adding a low dose of ketamine to an opioid, especially morphine, seems to improve pain relief. However, combination treatment with potentially addictive drugs such as ketamine and opioid in a patient group with normal life expectancy and where treatment may be long term or repeated multiple times may cause problems in susceptible individuals.

## 5. Conclusions

Ketamine is a drug with complex mechanisms of action and many properties which make it interesting for pain management. However, treatment regimens differ widely and there are concerns regarding adverse effects. High doses of ketamine are reported to cause a range of adverse effects and should be avoided. Ketamine has low oral availability, and oral administration seems to be associated with a high rate of adverse effects. Spinal and epidural routes are not recommended because of issues of neurotoxicity. Although ketamine is a drug of addiction, safety data regarding long-term and/or intermittent treatment are lacking.

There is good evidence that ketamine in the perioperative setting reduces pain scores and opioid requirements. Adverse effects are mild or absent, and perioperative ketamine may decrease postoperative nausea and vomiting. It seems most beneficial for surgery associated with high levels of postoperative pain.

The evidence for the use of ketamine in palliative care is limited, and it is not possible to recommend any specific treatment regimen. However, despite the limited evidence, a trial of low-dose ketamine, adjuvant to opioid (morphine), may be warranted in refractory cancer pain or pain in palliative care.

The evidence regarding ketamine for chronic noncancer pain is extremely limited, and there is a lack of safety data concerning long-term or repeated treatments. Importantly, there seems to be no strong evidence for the current widespread use of intermittent ketamine infusions.

## Disclosures

R.F. Bell has nothing to disclose. E.A. Kalso reports personal fees from Pierre Fabre and personal fees from Grunenthal, outside the submitted work.

## Appendix A. Supplemental digital content

Supplemental digital content associated with this article can be found online at http://links.lww.com/PR9/A25 and http://links.lww.com/PR9/A26.

## References

[1] Abdallah CG Adams TG Kelmendi B Esterlis I Sanacora G Krystal JH Ketamine's mechanism of action: a path to rapid-acting antidepressants. Depress Anxiety 2016;33:689–97. 27062302 10.1002/da.22501 PMC4961540

[2] Abdallah CG Averill CL Salas R Averill LA Baldwin PR Krystal JH Mathew SJ Mathalon DH Prefrontal connectivity and glutamate transmission: relevance to depression pathophysiology and ketamine treatment. Biol Psychiatry Cogn Neurosci Neuroimaging 2017;2:566–74. 29034354 10.1016/j.bpsc.2017.04.006 PMC5635826

[3] Amr YM Multi-day low dose ketamine infusion as adjuvant to oral gabapentin in spinal cord injury related chronic pain: a prospective, randomized, double blind trial. Pain Physician 2010;13:245–49. 20495588

[4] Assouline B Tramèr MR Kreienbühl L Elia N Benefit and harm of adding ketamine to an opioid in a patient-controlled analgesia device for the control of postoperative pain: systematic review and meta-analyses of randomized controlled trials with trial sequential analyses. PAIN 2016;157:2854–64. 27780181 10.1097/j.pain.0000000000000705

[5] Available at: ketamineclinicsdirectory.com. Accessed March 27, 2018.

[6] Azari P Lindsay DR Briones D Clarke C Buchheit T Pyati S Efficacy and safety of ketamine in patients with complex regional pain syndrome: a systematic review. CNS Drugs 2012;26:215–28. 22136149 10.2165/11595200-000000000-00000

[7] Bell RF Low-dose subcutaneous ketamine infusion and morphine tolerance. PAIN 1999;83:101–3. 10506678 10.1016/s0304-3959(99)00096-2

[8] Bell RF Dahl JB Moore RA Kalso EA Perioperative ketamine for acute postoperative pain. Cochrane Database Syst Rev 2006:CD004603. 16437490 10.1002/14651858.CD004603.pub2

[9] Bell RF Eccleston C Kalso EA Ketamine as an adjuvant to opioids for cancer pain. Cochrane Database Syst Rev 2017;6:CD003351. 28657160 10.1002/14651858.CD003351.pub3 PMC6481583

[10] Bell RF Jaksch W Kalso EA Interpreting the evidence: reply to Spruyt et al. J Pain Symptom Manage 2014;47:e2–e4. 10.1016/j.jpainsymman.2013.12.230 24679554

[11] Bell RF Moore RA Intravenous ketamine for CRPS: making too much of too little? PAIN 2010;150:10–11. 20347222 10.1016/j.pain.2010.03.014

[12] Bonnet U Long-term ketamine self-injections in major depressive disorder: focus on tolerance in ketamine's antidepressant response and the development of ketamine addiction. J Psychoactive Drugs 2015;47:276–85. 26317449 10.1080/02791072.2015.1072653

[13] Bredlau AL Thakur R Korones DN Dworkin RH Ketamine for pain in adults and children with cancer: a systematic review and synthesis of the literature. Pain Med 2013;14:1505–17. 23915253 10.1111/pme.12182

[14] Brinck ECV Tiippana E Heesen M Bell RF Straube S Moore RAS Kontinen V Perioperative intravenous ketamine for acute postoperative pain. Cochrane Database Syst Rev 2018. Under review. 10.1002/14651858.CD012033.pub4 PMC6360925 30570761

[15] Dale O Somogyi AA Li Y Sullivan T Shavit Y Does intraoperative ketamine attenuate inflammatory reactivity following surgery? A systematic review and meta-analysis. Anesth Analg 2012;115:934–43. 22826531 10.1213/ANE.0b013e3182662e30

[16] Elia N Tramèr MR Ketamine and postoperative pain–a quantitative systematic review of randomised trials. PAIN 2005;113:61–70. 15621365 10.1016/j.pain.2004.09.036

[17] Grégoire MC MacLellan DL Finley GA A pediatric case of ketamine-associated cystitis (Letter-to-the-Editor RE: Shahani R, Streutker C, Dickson B, et al: ketamine-associated ulcerative cystitis: a new clinical entity. Urology 69:810–812, 2007). Urology 2008;71:1232–3. 18455768 10.1016/j.urology.2007.11.141

[18] Hardy J Quinn S Fazekas B Plummer J Eckermann S Agar M Spruyt O Rowett D Currow DC Randomized, double-blind, placebo-controlled study to assess the efficacy and toxicity of subcutaneous ketamine in the management of cancer pain. J Clin Oncol 2012;30:3611–17. 22965960 10.1200/JCO.2012.42.1081

[19] Hocking G Cousins MJ Ketamine in chronic pain management: an evidence-based review. Anesth Analg 2003;97:1730–39. 14633551 10.1213/01.ANE.0000086618.28845.9B

[20] Karpinski N Dunn J Hansen L Masliah E Subpial vacuolar myelopathy after intrathecal ketamine: report of a case. PAIN 1997;73:103–5. 9414063 10.1016/s0304-3959(97)00068-7

[21] Laskowski K Stirling A McKay WP Lim HJ A systematic review of intravenous ketamine for postoperative analgesia. Can J Anaesth 2011;58:911–23. 21773855 10.1007/s12630-011-9560-0

[22] Laulin JP Maurette P Corcuff JB Rivat C Chauvin M Simonnet G The role of ketamine in preventing fentanyl-induced hyperalgesia and subsequent acute morphine tolerance. Anesth Analg 2002;94:1263–69. 11973202 10.1097/00000539-200205000-00040

[23] Li Y Jackson KA Slon B Hardy JR Franco M William L Poon P Coller JK Hutchinson MR Currow DC Somogyi AA CYP2B6*6 allele and age substantially reduce steady-state ketamine clearance in chronic pain patients: impact on adverse effects. Br J Clin Pharmacol 2015;80:276–84. 25702819 10.1111/bcp.12614 PMC4541975

[24] Lilius TO Jokinen V Neuvonen MS Niemi M Kalso EA Rauhala PV Ketamine coadministration attenuates morphine tolerance and leads to increased brain concentrations of both drugs in the rat. Br J Pharmacol 2015;172:2799–813. 25297798 10.1111/bph.12974 PMC4439876

[25] Lilius T Kangas E Niemi M Rauhala P Kalso E Ketamine and norketamine attenuate oxycodone tolerance markedly less than that of morphine: from behaviour to drug availability. Br J Anaesth 2018;120:818–26. 29576122 10.1016/j.bja.2017.11.081

[26] Lilius TO Viisanen H Jokinen V Niemi M Kalso EA Rauhala PV Interactions of (2S,6S;2R,6R)-hydroxynorketamine, a secondary metabolite of (R,S)-ketamine, with morphine. Basic Clin Pharmacol Toxicol 2018;122:481–88. 29171155 10.1111/bcpt.12941

[27] Liu SYW Ng SKK Tam YH Yee SCH Lai FPT Hong CYL Chiu PWY Ng EKW Ng CF Clinical pattern and prevalence of upper gastrointestinal toxicity in patients abusing ketamine. J Dig Dis 2017;18:504–10. 28749602 10.1111/1751-2980.12512

[28] Lu W Wang L Wo C Yao J Ketamine attenuates osteoarthritis of the knee via modulation of inflammatory responses in a rabbit model. Mol Med Rep 2016;13:5013–20. 27109206 10.3892/mmr.2016.5164 PMC4878578

[29] Mayell A Natusch D Anosmia–a potential complication of intranasal ketamine. Anaesthesia 2009;64:457–58. 10.1111/j.1365-2044.2009.05911.x 19317728

[30] McNicol ED Schumann R Haroutounian S A systematic review and meta-analysis of ketamine for the prevention of persistent post-surgical pain. Acta Anaesthesiol Scand 2014;58:1199–213. 25060512 10.1111/aas.12377

[31] Mercadante S Arcuri E Tirelli W Casuccio A Analgesic effect of intravenous ketamine in cancer patients on morphine therapy: a randomized, controlled, double-blind, crossover, double-dose study. J Pain Symptom Manage 2000;20:246–52. 11027905 10.1016/s0885-3924(00)00194-9

[32] Michelet D Brasher C Horlin AL Bellon M Julien-Marsollier F Vacher T Pontone S Dahmani S Ketamine for chronic non-cancer pain: a meta-analysis and trial sequential analysis of randomized controlled trials. Eur J Pain 2018;22:632–46. 29178663 10.1002/ejp.1153

[33] Mion G Villevieille T Ketamine pharmacology: an update (pharmacodynamics and molecular aspects, recent findings). CNS Neurosci Ther 2013;19:370–80. 23575437 10.1111/cns.12099 PMC6493357

[34] Mitchell AC Generalized hyperalgesia and allodynia following abrupt cessation of subcutaneous ketamine infusion. Palliat Med 1999;13:427–28. 10659115 10.1191/026921699667559279

[35] Mitchell AC Fallon MT A single infusion of intravenous ketamine improves pain relief in patients with critical limb ischaemia: results of a double blind randomised controlled trial. PAIN 2002;97:275–81. 12044624 10.1016/S0304-3959(02)00033-7

[36] Moyse DW Kaye AD Diaz JH Qadri MY Lindsay D Pyati S Perioperative ketamine administration for thoracotomy pain. Pain Physician 2017;20:173–84. 28339431

[37] Murphy EJ Acute pain management pharmacology for the patient with concurrent renal or hepatic disease. Anaesth Intensive Care 2005;33:311–22. 15973913 10.1177/0310057X0503300306

[38] Noppers IM Niesters M Aarts LP Bauer MC Drewes AM Dahan A Sarton EY Drug-induced liver injury following a repeated course of ketamine treatment for chronic pain in CRPS type 1 patients: a report of 3 cases. PAIN 2011;152:2173–78. 21546160 10.1016/j.pain.2011.03.026

[39] Olofsen E Noppers I Niesters M Kharasch E Aarts L Sarton E Dahan A Estimation of the contribution of norketamine to ketamine-induced acute pain relief and neurocognitive impairment in healthy volunteers. Anesthesiology 2012;117:353–64. 22692377 10.1097/ALN.0b013e31825b6c91 PMC3406234

[40] Oshima E Tei K Kayazawa H Urabe N Continuous subcutaneous injection of ketamine for cancer pain. Can J Anaesth 1990;37:385–6. 10.1007/BF03005598 2322977

[41] Peltoniemi MA Hagelberg NM Olkkola KT Saari TI Ketamine: a review of clinical pharmacokinetics and pharmacodynamics in anesthesia and pain therapy. Clin Pharmacokinet 2016;55:1059–77. 27028535 10.1007/s40262-016-0383-6

[42] Pendi A Field R Farhan SD Eichler M Bederman SS Perioperative ketamine for analgesia in spine surgery: a meta-analysis of randomized controlled trials. Spine (Phila Pa 1976) 2018;43:E299–E307. 28700455 10.1097/BRS.0000000000002318 PMC5846492

[43] Rigo FK Trevisan G Godoy MC Rossato MF Dalmolin GD Silva MA Menezes MS Caumo W Ferreira J Management of neuropathic chronic pain with methadone combined with ketamine: a randomized, double blind, active-controlled clinical trial. Pain Physician 2017;20:207–15. 28339433

[44] Schwartzman RJ Alexander GM Grothusen JR Paylor T Reichenberger E Perreault M Outpatient intravenous ketamine for the treatment of complex regional pain syndrome: a double-blind placebo controlled study. PAIN 2009;147:107–15. 19783371 10.1016/j.pain.2009.08.015

[45] Sigtermans MJ van Hilten JJ Bauer MC Arbous MS Marinus J Sarton EY Dahan A Ketamine produces effective and long-term pain relief in patients with Complex Regional Pain Syndrome Type 1. PAIN 2009;145:304–11. 19604642 10.1016/j.pain.2009.06.023

[46] Sleigh J Harvey M Voss L Denny B Ketamine—more mechanisms of action than just NMDA blockade. Trends Anaesth Crit Care 2014;4:76–81.

[47] Storr TM Quibell R Can ketamine prescribed for pain cause damage to the urinary tract? Palliat Med 2009;23:670–72. 19648225 10.1177/0269216309106828

[48] Stotz M Oehen HP Gerber H Histological findings after long-term infusion of intrathecal ketamine for chronic pain: a case report. J Pain Symptom Manage 1999;18:223–28. 10517045 10.1016/s0885-3924(99)00069-x

[49] Trujillo KA Akil H Inhibition of morphine tolerance and dependence by the NMDA receptor antagonist MK-801. Science 1991;251:85–7. 1824728 10.1126/science.1824728

[50] Trujillo KA Zamora JJ Warmoth KP Increased response to ketamine following treatment at long intervals: implications for intermittent use. Biol Psychiatry 2008;63:178–83. 17568566 10.1016/j.biopsych.2007.02.014

[51] US Ketamine clinics continue to mushroom with no regulation. Available at: Medscape.com. Accessed April 1, 2018.

[52] Van Hecke O Guthrie B Oral ketamine analgesia in chronic pain and problematic rise in blood pressure. BMJ Case Rep 2014;2014:bcr2014207836. 10.1136/bcr-2014-207836 PMC4248103 25425255

[53] Vranken JH Dijkgraaf MG Kruis MR van Dasselaar NT van der Vegt MH Iontophoretic administration of S(+)-ketamine in patients with intractable central pain: a placebo-controlled trial. PAIN 2005;118:224–31. 16202531 10.1016/j.pain.2005.08.020

[54] Vranken JH Troost D Wegener JT Kruis MR van der Vegt MH Neuropathological findings after continuous intrathecal administration of S(+) ketamine for the management of neuropathic cancer pain. PAIN 2005;117:231–35. 16098665 10.1016/j.pain.2005.06.014

[55] Wang L Johnston B Kaushal A Cheng D Zhu F Martin J Ketamine added to morphine or hydromorphone patient-controlled analgesia for acute postoperative pain in adults: a systematic review and meta-analysis of randomized trials. Can J Anaesth 2016;63:311–25. 26659198 10.1007/s12630-015-0551-4

[56] Ward J Standage C Angina pain precipitated by a continuous subcutaneous infusion of ketamine. J Pain Symptom Manage 2003;25:6–7. 12565182 10.1016/s0885-3924(02)00603-6

[57] Yaksh TL Fisher CJ Hockman TM Wiese AJ Current and future issues in the development of spinal agents for the management of pain. Curr Neuropharmacol 2017;15:232–59. 26861470 10.2174/1570159X14666160307145542 PMC5412694

[58] Yang CY Wong CS Chang JY Ho ST Intrathecal ketamine reduces morphine requirements in patients with terminal cancer pain. Can J Anaesth 1996;43:379–83. 8697554 10.1007/BF03011718

[59] Yang L Zhang J Zhang Z Zhang C Zhao D Li J Preemptive analgesia effects of ketamine in patients undergoing surgery. A meta-analysis. Acta Cir Bras 2014;29:819–25. 25517496 10.1590/S0102-86502014001900009

[60] Ye F Wu Y Zhou C Effect of intravenous ketamine for postoperative analgesia in patients undergoing laparoscopic cholecystectomy: a meta-analysis. Medicine (Baltimore) 2017;96:e9147. 29390443 10.1097/MD.0000000000009147 PMC5758145

[61] Zhao J Wang Y Wang D The effect of ketamine infusion in the treatment of complex regional pain syndrome: a systemic review and meta-analysis. Curr Pain Headache Rep 2018;22:12. 29404715 10.1007/s11916-018-0664-x

[62] Zhu W Ding Z Zhang Y Shi J Hashimoto K Lu L Risks associated with misuse of ketamine as a rapid-acting antidepressant. Neurosci Bull 2016;32:557–64. 27878517 10.1007/s12264-016-0081-2 PMC5567488

[63] Zhu J Xie H Zhang L Chang L Chen P Efficiency and safety of ketamine for pain relief after laparoscopic cholecystectomy: a meta-analysis from randomized controlled trials. Int J Surg 2018;49:1–9. 29175493 10.1016/j.ijsu.2017.11.031

